# Comparison of The Thickness of Free Anterolateral Thigh Flap in Different Fascial Planes: Clinical Results of Subfascial and Superficial Fat Flap

**DOI:** 10.1055/s-0043-1772586

**Published:** 2023-12-01

**Authors:** Yavuz Tuluy, Zülfükar Ulaş Bali, Merve Özkaya Ünsal, Aziz Parspancı, Levent Yoleri, Çağla Çiçek, Gaye Taylan Filinte

**Affiliations:** 1 Department of Plastic, Reconstructive and Aesthetic Surgery, Turgutlu State Hospital, Manisa, Turkey; 2 Plastic, Reconstructive and Aesthetic Surgery, İstanbul, Turkey; 3 Department of Plastic, Reconstructive and Aesthetic Surgery, İzmir, Turkey; 4 Department of Plastic Reconstructive and Aesthetic Surgery, Bayburt State Hospital, Bayburt, Turkey; 5 Department of Plastic Reconstructive and Aesthetic Surgery, Manisa Celal Bayar University Faculty of Medicine, Manisa, Turkey; 6 Department of Plastic Reconstructive and Aesthetic Surgery, İstanbul Kartal Dr. Lütfi Kırdar City Hospital, İstanbul, Turkey

**Keywords:** anterolateral thigh flap, reconstruction, free flap, superficial fat flap

## Abstract

**Background** The anterolateral thigh (ALT) flap is a preferred option in the reconstruction of a wide variety of defects, enabling multiple tissue components and thicknesses.

**Methods** This study was conducted to investigate the correlation of the thickness of the traditional subfascial ALT flap and superficial fat flap with age, gender, and body mass index (BMI). A total of 42 patients (28 males and 14 females) were included in the study.

**Results** Mean age was 50.2 (range, 16–75) years and mean BMI was 24.68 ± 4.02 (range, 16.5–34.7) kg/m ^2^ . The subfascial flap thickness was significantly thinner in male patients (16.07 ± 2.77 mm) than in female patients (24.07 ± 3.93 mm; *p* < 0.05), whereas no significant difference was found between male (4.28 ± 1.15 mm) and female patients (4.85 ± 1.09 mm) regarding superficial fat flap thickness ( *p* = 0.13). The thickness of both flaps had a positive correlation with BMI, and the strongest correlation was found for subfascial ALT thickness in female patients ( *r* = 0.81). Age had no effect on both flap thickness measurements. The anterior thigh is thicker in women than in men, although it varies according to BMI. This shows that flap elevation is important in the superthin plane, especially if a thin flap is desired in female patients in defect reconstruction with the ALT flap. Thus, a single-stage reconstruction is achieved without the need for a defatting procedure after subfascial dissection or a second defatting procedure 3 to 6 months later.

**Conclusion** The appropriate ALT flap plane should be selected considering the gender and BMI of the patient.

## Introduction

Since its first description by Song et al in 1984, the anterolateral thigh (ALT) flap has gained popularity among surgeons for multiple reasons, 1 namely including low donor site morbidity, preservation of major vascular structures, ability to be performed via two-team approach, and consistent vascular anatomy. 2 3 4 5 6 7 8 9 Although it is designed as a fasciocutaneous flap, the contents and thickness of ALT can be modulated and thus it can be adapted to many clinical conditions. 10 11 Fascia-only flap, suprafascial flaps, and thinned flaps are other techniques investigated by numerous studies discussing modifications in ALT flaps. 11 12 13 14 15 16 17 Although the thickness of suprafascial and superficial fat flaps are adjustable and these flaps offer the advantage of reconstruction of shallow defects, particularly in the head and neck, traditional subfascial flaps can be combined with underlying muscular structures if bulkiness is needed. 2 15 18

ALT flap can vary in adipose thickness, and it has been reported that female and obese individuals tend to have more bulky flaps. 19 However, the effects of age, gender, and body mass index (BMI) of the patient on ALT flaps remain unclear. The aim of this study was to compare the thickness of the traditional subfascial ALT flap with that of the superficial fat flap in the same patient group and to investigate the correlation between flap thickness and age, gender, and BMI.

## Methods



**Video 1:** Superficial fat ALT flap elevation. ALT, anterolateral thigh.


**Fig. 1:**
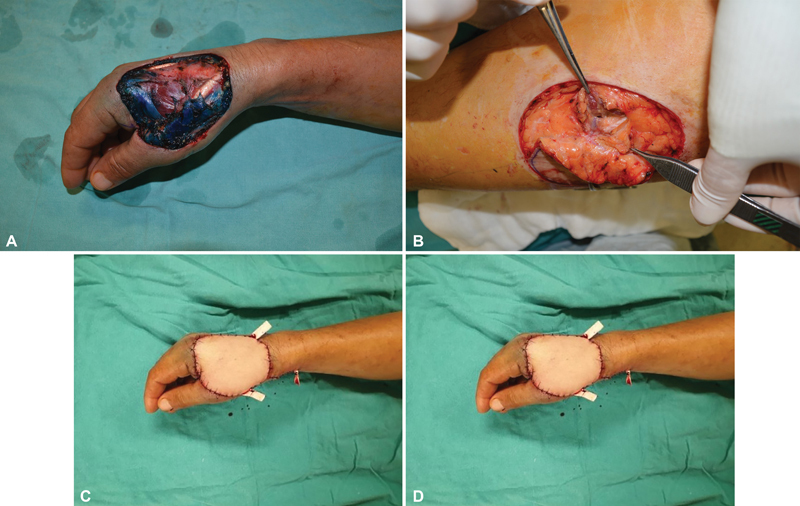
(A) A 58-year-old male, defect on his right hand after squamous cell skin cancer excision. (B) Flap elevation, subfascial thickness: 14 mm, superficial fat flap thickness: 5 mm, BMI: 25.8 kg/m ^2^ . (C) Early postoperative view. (D) Postoperative 29-month view.

**Fig. 2:**
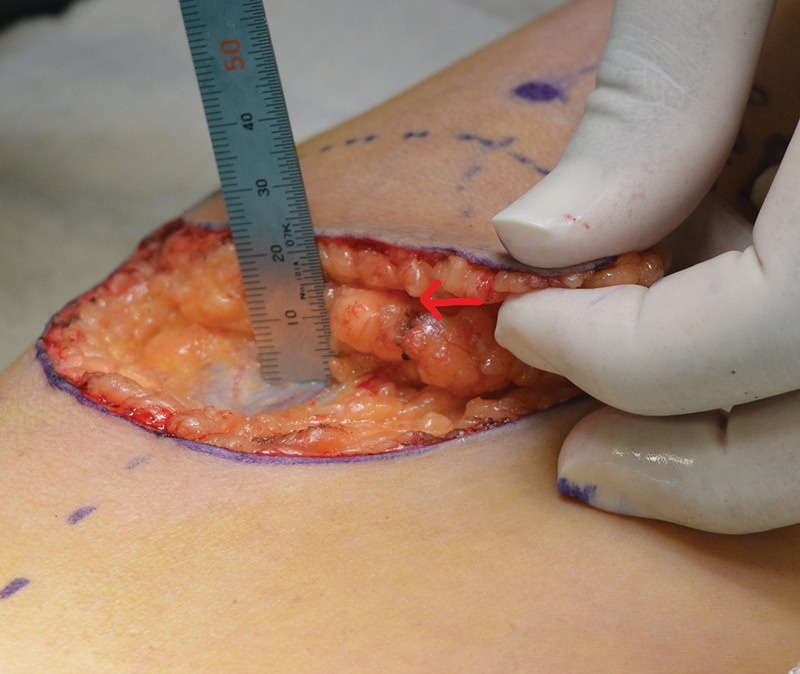
A 44-year-old female, operated for trauma to right foot ankle. Subfascial thickness: 23 mm, superficial fat flap thickness: 6 mm, BMI: 23.9 kg/m ^2^ . Red arrow shows the superficial fascia. BMI, body mass index.

**Fig. 3:**
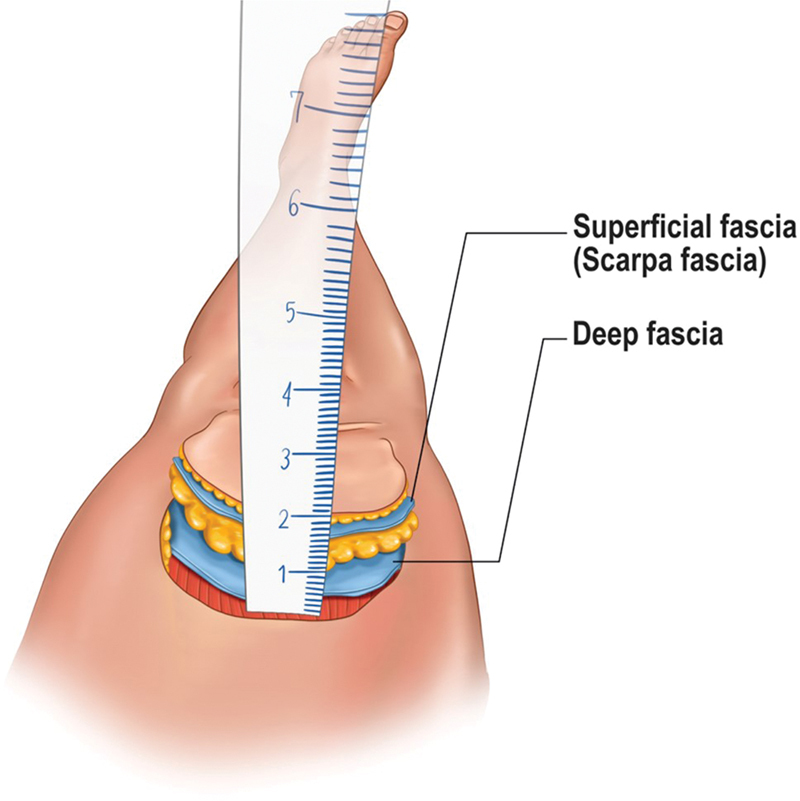
The image showing the fascial planes of superficial fat and subfascial ALT flaps. The measurements were done on the proximal part of the flap through the same point and both numbers were noted for each patient. ALT, anterolateral thigh.

The study was approved by İstanbul Kartal Dr. Lütfi Kırdar Educational and Training Hospital Institutional Review Board (Ref. No. 514/194/9). The prospective study included 42 patients (28 male and 14 females; age range, 16–75 years) who underwent defect reconstruction with the ALT flap in Manisa Celal Bayar University Hospital and İstanbul Kartal Dr. Lütfi Kırdar Educational and Training Hospital between June 2019 and October 2020. Informed consent forms were obtained from the patients for the operation, use of data, and photographs. Age, gender, etiology, flap dimensions and thicknesses BMI, and complications were recorded for each patient **(** Tables 1 and 2 **)** . The operation was performed under general anesthesia. Preoperative markings of ALT perforators were made based on the anatomical landmarks described by Wei et al. 2 A proximal incision was performed deep to the fascia lata in the deep plane for traditional subfascial ALT flap, and then, the flap was raised above the superficial fascia plane for the superficial fat flap as described in the literature ( Video 1 ). 20 Scarpa fascia is a very thin structure and may not be seen clearly in every patient. The transition point of thin and thick fat lobules can be evaluated more clearly with loupe magnification. The scarpa fascia is in the region of this transition. We used monopolar cautery for dissecting superficial planes and bipolar cautery and scissors for the dissection of the pedicle. Pedicle dissection was started from the distal and continued proximally. Type 1 and type 2 pedicles described by Schaverien et al were chosen for dissection. 21 To prevent pedicle injury, approximately 2 cm of fatty tissue around the pedicle was included in the flap ( Fig.1A – **D** ). The flap thickness in the subfascial and superficial scarpa fascia planes were measured perioperatively on the same point of the flap using a caliper ( Fig. 2 ). In all patients, the flap was measured on the proximal part, that is, its thickest part, and two measurements were recorded for each flap ( Fig. 3 ). The surgical procedure was continued in its natural course in all patients. Perioperative 5,000 IU heparin intravenous was administered to each patient.

**Table 1:** Demographic characteristics and the etiology of the patients

Patient	Gender	Age	Etiology
1	F	57	Diabetic foot ulcer
2	F	48	Left submandibular defect (mandibular osteomyelitis)
3	F	70	Diabetic foot ulcer
4	F	55	Lower lip cancer
5	F	44	Trauma (right foot ankle)
6	F	16	Trauma (left cruris)
7	F	51	Liposarcoma (left cruris)
8	F	53	Trauma (right cruris)
9	F	44	Scalp defect (total scalp avulsion)
10	F	75	Skin cancer (right medial malleolus)
11	F	44	Trauma (right cruris lateral)
12	F	63	Skin cancer (left cruris anterior)
13	F	60	Burn defect (right foot)
14	F	65	Skin cancer (scalp)
15	M	44	Left ankle
16	M	38	Burn contracture (left foot)
17	M	62	Trauma (right foot)
18	M	51	Lower lip cancer
19	M	45	Gunshot (lower lip defect)
20	M	66	Lower lip cancer
21	M	57	Skin cancer (left hand)
22	M	40	Trauma (left cruris anterior)
23	M	22	Trauma (left cruris anterior)
24	M	29	Trauma (right cruris anterior)
25	M	26	Trauma (left cruris anterior)
26	M	47	Lower lip cancer
27	M	71	Skin cancer (left neck)
28	M	35	Trauma (left ankle)
29	M	68	Tongue cancer
30	M	37	Gunshot (lower lip and left cheek defect)
31	M	70	Skin cancer (left temporal area)
32	M	58	Skin cancer (right hand dorsum)
33	M	53	Diabetic foot ulcer
34	M	46	Trauma (right cruris lateral)
35	M	54	Skin cancer (left hand)
36	M	48	Trauma (left cruris)
37	M	56	Diabetic foot ulcer
38	M	42	Vascular malformation (right hand)
39	M	33	Trauma (right cruris lateral)
40	M	62	Skin cancer (left foot)
41	M	51	Lower lip cancer
42	M	53	Skin cancer (scalp)

**Table 2:** Flap characteristics and postoperative results

Patient	Subfascial thickness (mm)	Superficial fat flap thickness (mm)	Flap dimensions (mm ^2^ )	Body mass index	Follow-up	Complication
1	24	6	9 × 6	25.5	18	Partial necrosis (secondary intention)
2	30	7	12 × 8	33.7	23	None
3	24	6	9 × 6	25.5	33	Total necrosis (skin graft)
4	20	4	11 × 7	20.3	28	None
5	23	6	12 × 8	23.9	18	None
6	20	4	6 × 5	21.1	32	None
7	26	4	16 × 8	24.9	26	None
8	29	5	20 × 10	28.5	31	Wound dehiscence (secondary intention)
9	23	5	28 × 26	27.1	34	Wound dehiscence (secondary intention)
10	19	3	20 × 12	21.7	32	None
11	18	3	16 × 9	21.5	16	None
12	26	5	9 × 7	25.1	32	None
13	28	6	25 × 13	25.7	15	Total necrosis (reconstruction with ALT)
14	27	5	12 × 6	24.2	33	None
15	18	4	10 × 8	30.2	22	None
16	13	4	8 × 6	20.5	38	None
17	14	3	12 × 6	22.2	23	None
18	13	5	10 × 6	19.8	26	None
19	15	4	8 × 6	21.8	32	None
20	13	3	8 × 6	21.4	26	Neck hematoma
21	15	5	4 × 3	24.5	32	None
22	14	3	10 × 6	24.7	26	None
23	14	4	15 × 9	23.6	22	None
24	18	4	12 × 8	24.7	27	Partial necrosis (secondary intention)
25	17	6	10 × 6	29.4	24	None
26	21	6	10 × 6	32.5	30	None
27	18	4	30 × 12	29.3	36	None
28	14	5	18 × 10	23.6	36	None
29	16	3	8 × 6	22.3	26	None
30	14	3	12 × 8	21.5	22	None
31	12	3	8 × 6	19.4	16	None
32	14	5	10 × 7	25.8	29	None
33	21	3	12 × 6	22.9	25	Partial necrosis (secondary intention)
34	14	3	25 × 12	23.1	22	None
35	19	5	5 × 3	24.7	28	None
36	15	3	16 × 11	24.2	16	None
37	20	6	12 × 8	34.7	33	None
38	13	3	8 × 6	16.5	24	None
39	20	6	16 × 8	32.9	16	None
40	18	6	17 × 12	26.4	23	None
41	20	5	8 × 6	24.9	28	Neck hematoma
42	17	5	10 × 8	20.6	18	None

### Statistical Analysis

Data were analyzed using SPSS for Windows version 21 (Armonk, NY: IBM Corp.). The normality of continuous variables (both for main groups and subgroups) was analyzed using the Shapiro–Wilk test. Descriptive features were expressed as arithmetic mean, standard deviation (SD), minimum, and maximum. Independent samples *t* -test, Pearson correlation coefficient, and partial correlation were used as statistical tests. The statistical significance level (alpha) was set to 0.05.

## Results

The 42 patients comprised 28 (66.7%) men and 14 (33.3%) women with a mean age of 50.21  ± 13.65 (range, 16–75) years and a mean BMI of 24.68 ± 4.02 (range, 16.5–34.7) kg/m ^2^ ( Tables 2 and 3 ). Mean flap thickness was 18.73 ± 4.95 (range, 12–30) mm for the subfascial flap and 4.47 ± 1.15 (range 3–7) mm for the superficial fat flap. Flap dimensions ranged from 4 × 3 cm to 28 × 26 cm. Intravenous hydration was given for 2 days postoperatively, with oral intake closed and positive balance in all patients. Subcutaneous low-molecular-weight heparin was administered for 5 days. Afterward, oral 100 mg of acetylsalicylic acid was continued until the postoperative 3rd week. Partial flap necrosis developed in three patients, and total necrosis developed in two patients. One of the patients with total necrosis, defect was reconstructed with a skin graft and the other was reconstructed with a superficial fat flap from the opposite side. Partial and total flap necrosis was occurred due to the venous thrombosis. We checked the anastomosis and removed the thrombosis. All patients with partial necrosis recovered secondary. Two patients with hematoma in the neck region were explored again and bleeding was controlled. Although the hematoma was close to the recipient vessel area, no circulation problem was observed in the flaps. The patients were followed up for an average of 26 (15–38) months. There was no need for flap thinning in the follow-ups. We did not use any kind of flap compression postoperatively.

**Table 3:** Summary of demographic and clinical characteristics

	*n*	Age	Subfascial flap thickness	Superficial fat flap thickness	BMI
Male	28	48.71 ± 13.23	16.07 ± 2.77 mm	4.28 ± 1.15 mm	24.57 ± 4.34 kg/m ^2^
Female	14	53.21 ± 14.48	24.07 ± 3.93 mm	4.85 ± 1.09 mm	24.90 ± 3.45 kg/m ^2^
*p* -Value		0.320	0.0001	0.132	0.805

Abbreviation: BMI, body mass index.

No significant difference was found between male and female patients regarding BMI (24.57 ± 4.34 vs. 24.90 ± 3.45 kg/m ^2^ , respectively) and age (48.71 ± 13.23 vs. 53.21 ± 14.48 years; *p* = 0.80 and *p* = 0.32, respectively; Table 2 ).

Subfascial flap thickness was significantly lower in male patients (16.07 ± 2.77 mm) compared to female patients (24.07 ± 3.93 mm; *p* < 0.05). However, there was no significant difference between male and female patients regarding superficial fat flap thickness (4.28 ± 1.15 *vs.* 4.85 ± 1.09 mm, respectively; *p* = 0.13; Table 3 ).

BMI was positively correlated with both subfascial and superficial fat flap thickness ( *r* = 0.48 and *r* = 0.69, respectively) and this correlation was greater in female patients than in male patients both in subfascial and superficial fat flaps ( *r* = 0.78 and *r* = 0.67, respectively) ( Table 4 , Graphics 1 2 3 4 ). Nonetheless, these correlations were not influenced by age.

**Table 4:** Correlation of flap thicknesses with gender and BMI

	Subfascial flap thickness		Superthin flap thickness	
	*r*	*p* -Value	*r*	*p* -Value
Male	0.711	0.000	0.676	0.000
Female	0.812	0.000	0.785	0.001

Abbreviation: BMI, body mass index.

**Graphic 1:**
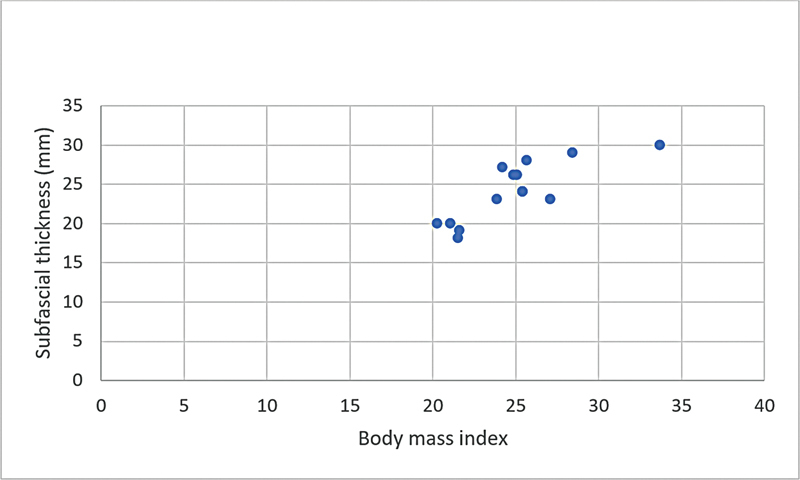
Correlation of subfascial flap thickness and body mass index in female patients.

**Graphic 2:**
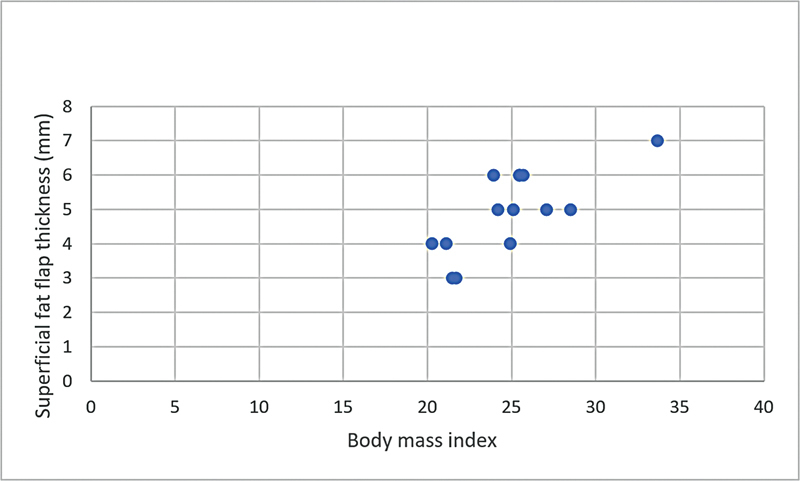
Correlation of superficial fat flap thickness and body mass index in female patients.

**Graphic 3:**
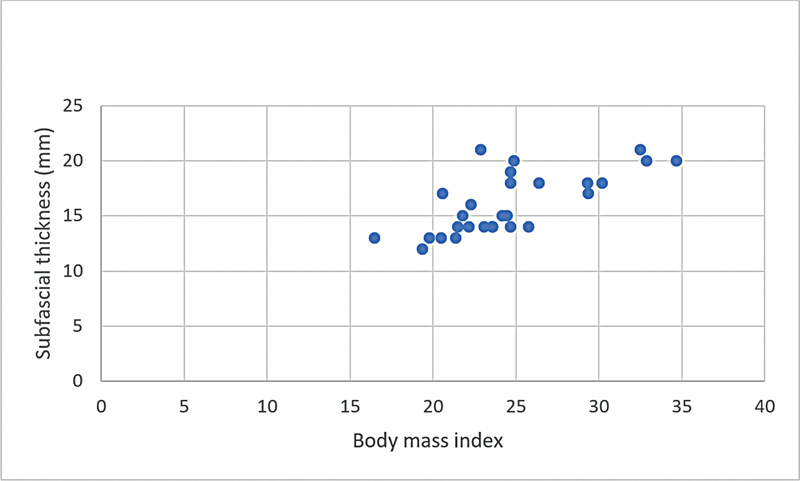
Correlation of subfascial flap thickness and body mass index in male patients.

**Graphic 4:**
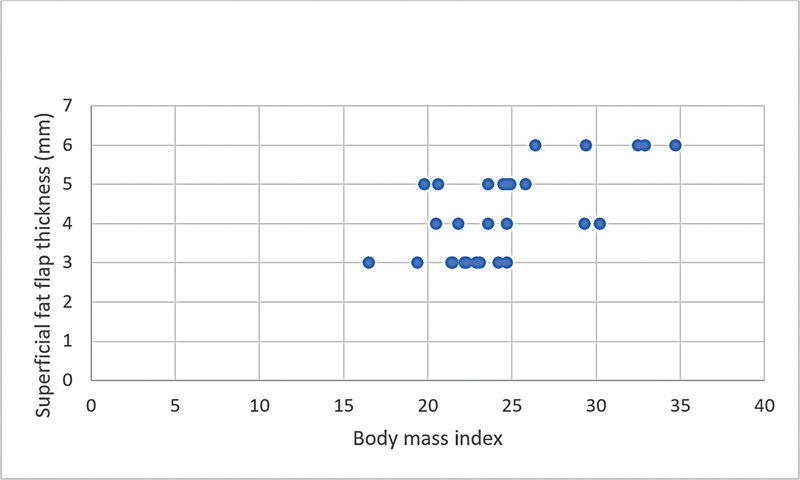
Correlation of superficial fat flap thickness and body mass index in male patients.

## Discussion

Achieving an optimal reconstruction using a free flap requires favorable functional and cosmetic outcomes. The ALT flap has become an increasingly popular technique mainly because its adaptability to numerous clinical conditions and variable tissue modifications fulfill the reconstruction needs. 2 A subfascial ALT flap results in a bulky appearance that may cause both functional (e.g., airway obstruction following reconstruction of tongue) and cosmetic (e.g., incompatibility with surrounding tissue) deficiency and may lead to debulking procedures. 2 7 18 20 By contrast, a thin ALT flap can be obtained by meticulous dissection to be performed by trimming the fat lobules after harvesting the flap. 6 17 However, thinning the flap safely requires experience and also has a risk of injuring the perforator during trimming and can be time-consuming. 11 14 15 22 Accordingly, a good understanding of the anterolateral region and the effects of personal factors on ALT flap is needed to avoid secondary surgeries.

Schaverien et al divided flap pedicles into three types. 21 In type 1, the perforator obliquely passes through the fatty tissue and extends to the subdermal plexus. In type 2, while the perforator extends to the subdermal plexus, it also gives horizontal branches in the suprafacial plane. In type 3, the perforator gives numerous horizontal branches in the fatty tissue in the suprafacial plane and then reaches to the subdermal plexus. In our study, flap harvest was performed by dissecting type 1 and type 2 perforators from distal to proximal. Since the pedicle course can be followed on the superficial fat flap, we can choose the appropriate pedicles. To reduce the risk of pedicle damage in flap elevation over a type 2 perforator, 1 to 2 cm wide fatty tissue around the pedicle is also included in the pedicle. Since it is possible to follow the perforator in the entire fatty tissue, we think that the superficial fat flap is more reliable than the ALT flap that is thinned after elevation in the subfascial plane. In patients with ALT flap dissection in the subfascial plane, if the dissection is performed over a type 3 perforator, the risk of pedicle damage and related venous and arterial insufficiency will be high after thinning. If a thin flap is needed, the ALT flap should be elevated accordingly. Perioperative defatting requires extra time and because it is done toward the end of the case, fatigue of the surgeon may increase the risk of pedicle damage. The thinning process can also be performed in the following months after the operation, but second surgical stress and risks await the patient. Flap surgery is a major surgery and most of the patients do not want to experience the same stress again. The economic costs of hospitalization for the second time should also be considered. Another advantage of a superficial fat flap is that ensuring the continuity of the fascia prevents muscle herniation and it provides better thigh contour.

In the literature, the thickness of the ALT flap has been studied using radiological and invasive methods and the thickness of subcutaneous fat in different parts of the anterior thigh region has also been explored. 19 23 Moreover, the proportion of the skin and hypodermis has been extensively studied via histological analysis. 24 In a previous study, the thickest part of the thigh region was shown to be the upper part and the thinnest region to be the lower part and no significant difference was found between the left and right anterior thigh regions with regard to the thickness of subcutaneous fat. 19 On the contrary, it is commonly known that female patients have more fatty subcutaneous anterior thigh tissue. 19 23 25 In line with the literature, our findings indicated that subfascial ALT flap thickness was higher in female patients than in male patients, whereas superficial fat flap thickness showed no significant difference between the genders.

In imaging-based studies, Hsu et al examined the anterolateral region radiologically and reported that the flap thickness was 9.8 ± 4.0 mm in the measurements made by ultrasonography (USG) from the proximal one-fourth part of the ALT between the skin and the deep fascia. 25 Nakayama et al evaluated a series of 31 patients from the eastern population and measured the flap area by USG without clearly determining the measurement area and reported the mean flap thickness as 7.3 ± 3.4 mm. 26 Clearly, there are significant differences between the studies using USG and our study regarding thickness measurements and the fact that the BMIs of the patients in the eastern population were lower than those of our patients may explain the difference in flap thicknesses. In addition, this difference could also be associated with the deformity caused by the pressure of the USG probe and with the changes in skin tension after flap harvesting. In some similar studies, a water bag type of medium was used in order to minimize the effect of the pressure of the probe during the measurements made with USG. 26 However, flap thickness may differ from its actual value. We consider that the measurements made after flap harvesting minimize these effects on the outcomes, and thus, we obtain more objective results.

Obesity increases the adiposity of the upper thigh, and the rising prevalence of obesity necessitates considering the patient's BMI in surgical planning. 15 It is known that the subfascial ALT flap thickness has a positive correlation with BMI. 23 25 Our results were consistent with the literature, and it was also revealed that both subfascial and superficial fat ALT flaps had a positive correlation with BMI. Doğan et al reported a stronger correlation between ALT and BMI in male patients compared to female patients via USG ( *r* = 0.66). 23 In our study, however, the correlation between the subfascial ALT flap thickness and BMI was stronger in female patients ( *r* = 0.81). In the study conducted by Suh et al, no statistically significant correlation was found between BMI and flap thickness in patients who underwent defect reconstruction with a superthin ALT flap. 27 The statistically significant positive correlation in our study may be due to ethnic differences.

In a study on flap thicknesses by Kim et al, it was stated that when the flap is elevated over the superficial fascia, approximately one-third of the subfascial thickness is thinned. 28 In our study, although it varies according to gender and BMI, the thickness of the elevated flap over the superficial fascia varies between 20 and 25% of the subfascial flap thickness. This difference may be due to ethnic differences as well as the indistinct superficial fascia in patients.

Gong et al preoperatively measured the traditional ALT flap thickness of 66 patients (47 male and 19 female) and, in a similar way to our study, reported that the traditional ALT thickness was highest in the upper part (women, 25.92 ± 7.05 mm; men, 15.23 ± 5.68 mm). 19 Additionally, Maruccia et al also published similar findings in 34 patients with similar ALT flap thickness (26.2 ± 5.2 mm). 13 Nevertheless, it was not possible to compare the correlation tests in these studies since the measurements were made in different subject groups. However, in the present study, since the thickness of both traditional ALT flaps and superficial fat flaps were measured in the flaps harvested on the same subject, we consider that it is more likely to demonstrate the effects of individual factors on flap thickness. In the study by Cha et al, thicknesses of thin, superthin, and ultrathin ALT flaps were compared, and no significant difference was observed between complication rates. 29 Also, it was observed that the deep and superficial fascia was located more deeply in women than in men. The fact that there is no significant difference between the complications increases the reliability of the superficial fat flap.

In our study, it was stated that a safe ALT flap dissection could be achieved by choosing the appropriate pedicle type considering the BMI. Since flap thickness is correlated with BMI, especially in female patients, this should be considered to obtain a good result both functionally and aesthetically. The small sample size is the limitation of the study, and the findings are needed to be compared with the large series in the future. The other limitation of the study is that patient satisfaction was not evaluated.
